# Supplementary material for: Evaluating evidence for co-geography in the Anopheles Plasmodium host-parasite system
Source: bioRxiv. 2023 Jul 17:2023.07.17.549405. Preprint. [Version 1] doi: 10.1101/2023.07.17.549405 (PMC10370088; doi:10.1101/2023.07.17.549405)
Supplement: Supplementary file 1 [file NIHPP2023.07.17.549405V1-supplement-1.pdf]

bioRxiv preprint doi: https://doi.org/10.1101/2023.07.17.549405; this version posted July 17, 2023. The copyright holder for this preprint (which was not certified by peer review) is the author/funder, who has granted bioRxiv a license to display the preprint in perpetuity. It is made available under aCC-BY 4.0 International license.

Supplementary material

Windowed analysis and uncertainty

While our analysis focused on trends across individual predictions in a dataset, we were also interested in how estimated uncertainty is affected by training data. By visualizing the spatial spread of predictions using different portions of the genome, windowed analysis can be used to assess the uncertainty in individual-level predictions (Battey et al., 2020a). When examining the windowed predictions for individuals in our simulated datasets, we observed a negative correlation between local density of training data and the spatial spread of an individual's predictions across genomic windows, with this trend becoming more pronounced as training sets became more imbalanced (Figure S3A). When trained on uniformly-sampled data (bias = 0.5) with a dispersal rate of 1.0, the relationship between spatial density of training data and spatial spread of windowed predictions for a test sample is weakly negatively correlated: $r = -.10, p = 0.02, n = 500$. With a training set bias of 0.9, the relationship is much stronger: $r = -.67, p = 2.8 \times 10^{-65}, n = 500$.

Effect of training set size

We also assessed how increasing the number of training samples affects `Locator`'s performance on spatially imbalanced datasets (Figure S3, panels B-D). Initial analysis on randomly sampled training sets had indicated that 450 training samples were sufficient for accurate inference of location (Battey et al., 2020a), but this number of training samples did not provide the same results when sampled unevenly from the landscape. Unsurprisingly, increasing training set size reduced the effects of training set imbalance, even in populations undergoing a high rate of dispersal (1.0 units per generation). While residuals are still biased - correlation between x-axis error and training set skew remains significant with 4,500 training samples ($r = 0.72, p = 3.92 \times 10^{-9}$) - the magnitude of these errors is reduced, likely reflecting the overall increase in training data.

Sample weighting hyperparameters

During our grid search, we observed an inverse relationship between the magnitude of bandwidth and λ parameters required to reduce residual bias when training with spatially imbalanced data. At high values of λ, low bandwidth resulted in reduced bias (as measured by error along the x-axis), while overall prediction accuracy remained unaffected (as measured by y-axis error and residual magnitude). As bandwidth was increased, the value of λ that resulted in reduced prediction bias while maintaining accuracy decreased consistently. These combinations form a diagonal across the parameter space, with `Locator` performing differently on either side of this threshold (Figure S4).

When both λ and bandwidth were low, sample weights were relatively uniform across the landscape, which had a negligible effect on training outcomes: in the space beneath optimal λ/bandwidth combinations, `Locator`'s predictions were similar to those from unweighted training runs (Figure S5 C). Above the optimal threshold, where both λ and bandwidth values are high, performance was more inconsistent: residuals were biased along the x-axis

bioRxiv preprint doi: https://doi.org/10.1101/2023.07.17.549405; this version posted July 17, 2023. The copyright holder for this preprint (which was not certified by peer review) is the author/funder, who has granted bioRxiv a license to display the preprint in perpetuity. It is made available under aCC-BY 4.0 International license.

and y-axis, and overall error magnitude increased well beyond that observed when training without sample weights (Figure S5 E). With these parameter combinations, the majority of samples were assigned extremely low weights, causing `Locator` to only focus on a few samples near the landscape boundaries during training and predict samples to be in some landscape midpoint.

Comparison to `SPASIBA` Prior to our introduction of `Locator`, the state-of-the-art statistical method for geographic assignment using genotype data was perhaps `SPASIBA` (Guillot et al., 2015). `SPASIBA` uses a clever implementation of a geostatistical model which learns smooth surfaces of allele frequencies over a landscape, and then predicts locations of unlabeled samples by maximizing their likelihood on this surface. We were interested to compare the performance of `SPASIBA` and `Locator` when using spatially imbalanced training data, in particular because biased training sets may affect traditional statistical and machine learning methods to a greater or lesser extent.

In Figure S6 we show results from `SPASIBA` applied to training sets of 10,000 SNPs drawn from samples of 450 individuals. In some cases we were only able to execute seven `SPASIBA` analyses for a given training set bias, as compared to ten `Locator` analyses, because jobs were killed after 30 days of execution. As with `Locator`, `SPASIBA`'s predictions were biased towards the right half of the landscape when run on imbalanced training sets. However, `SPASIBA`'s predictions suffered significant collapse towards the center of the sampling density in some cases, and often covered a small fraction of the landscape, with test individuals across a run all predicted to be in only a few unique locations. These results suggest that `SPASIBA` may struggle to a greater extent than `Locator` in the face of imbalanced training data, and more generally that biased training sets can influence prediction in traditional statistical as well as machine learning settings.

bioRxiv preprint doi: https://doi.org/10.1101/2023.07.17.549405; this version posted July 17, 2023. The copyright holder for this preprint (which was not certified by peer review) is the author/funder, who has granted bioRxiv a license to display the preprint in perpetuity. It is made available under aCC-BY 4.0 International license.

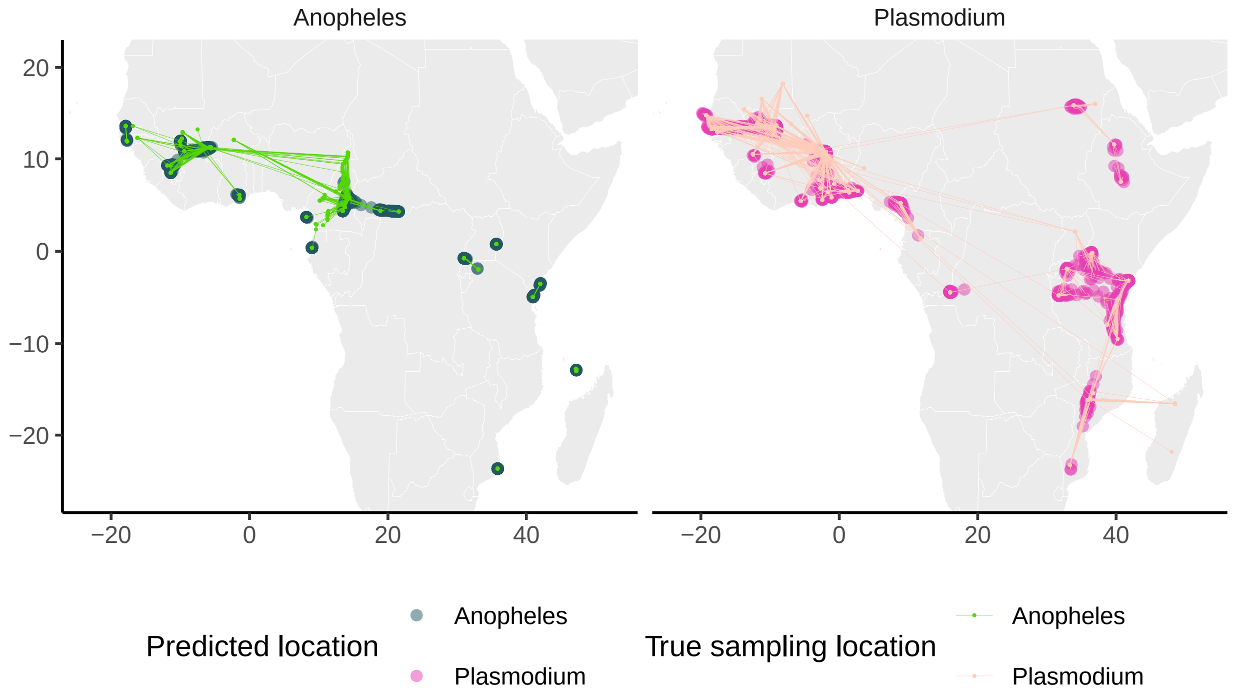

Figure S1: Predicted locations of all *A. gambiae* and *P. falciparum* samples. A sample's true location is connected to the geographic centroid of its genome-wide windowed predictions.

bioRxiv preprint doi: https://doi.org/10.1101/2023.07.17.549405; this version posted July 17, 2023. The copyright holder for this preprint (which was not certified by peer review) is the author/funder, who has granted bioRxiv a license to display the preprint in perpetuity. It is made available under aCC-BY 4.0 International license.

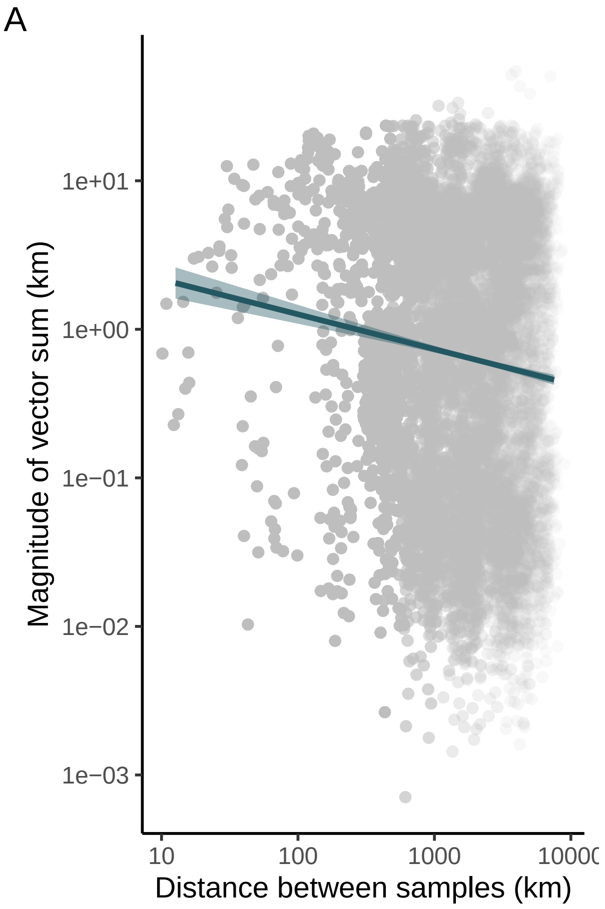

Figure S2: The magnitude of summed *A. gambiae* and *P. falciparum* residual vectors plotted against the distance between true sample locations. For both unweighted (A) and weighted (B) analyses, distance between samples is negatively correlated with the magnitude of summed residual vectors.

bioRxiv preprint doi: https://doi.org/10.1101/2023.07.17.549405; this version posted July 17, 2023. The copyright holder for this preprint (which was not certified by peer review) is the author/funder, who has granted bioRxiv a license to display the preprint in perpetuity. It is made available under aCC-BY 4.0 International license.

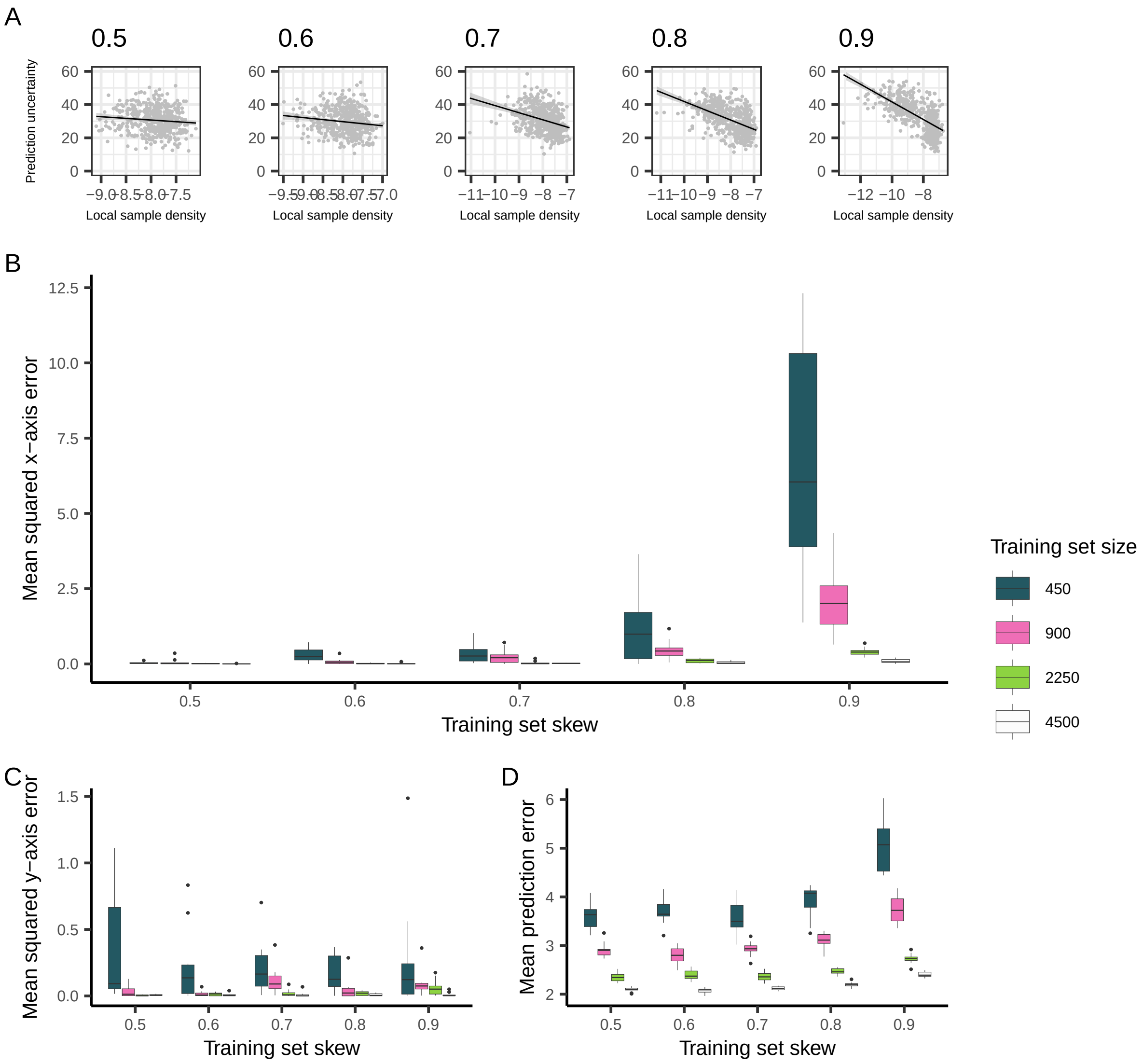

Figure S3: The effect of training set bias and size on `Locator` prediction error at a dispersal rate of $\sigma = 1.0$. (A) Spatial spread of per-individual windowed predictions as a function of local training set density at different levels of training set bias. (B) Mean per-run prediction error along the x axis as a function of training set bias, (C) mean per-run prediction error along the y axis, (D) mean per-run error.

bioRxiv preprint doi: https://doi.org/10.1101/2023.07.17.549405; this version posted July 17, 2023. The copyright holder for this preprint (which was not certified by peer review) is the author/funder, who has granted bioRxiv a license to display the preprint in perpetuity. It is made available under aCC-BY 4.0 International license.

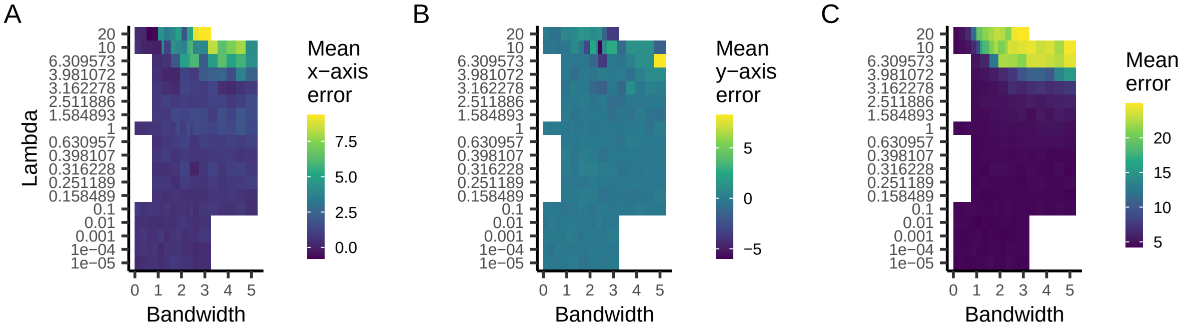

Figure S4: Results of grid search for λ and bandwidth parameters on $bias = 0.9$, $\sigma = 1.0$ training sets. (A) Mean x-axis error, (B) mean y-axis error, and (C) mean error for all tested combinations ofλ and bandwidth.

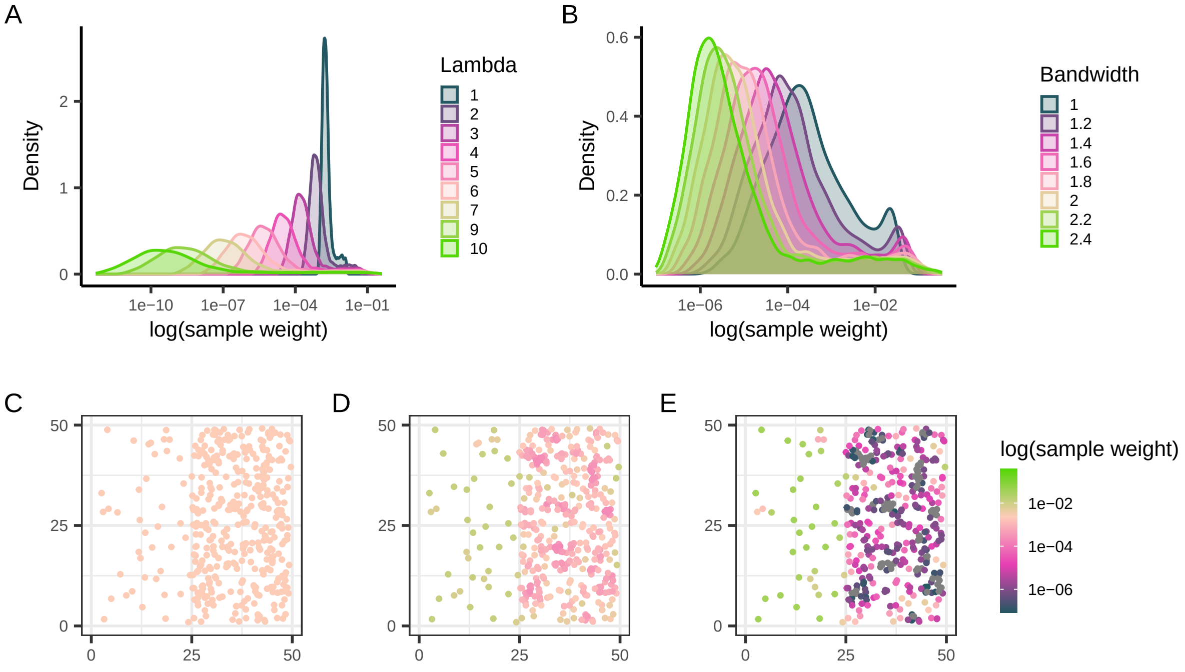

Figure S5: Bandwidth and λ parameters alter the distribution of sample weights. (A) Distribution of sample weights assigned to a given training set holding bandwidth constant and varying λ. (B) Distribution holding λ constant and varying bandwidth. (C, D, E) Biased training sets colored by their sample weights as λ increases.

bioRxiv preprint doi: https://doi.org/10.1101/2023.07.17.549405; this version posted July 17, 2023. The copyright holder for this preprint (which was not certified by peer review) is the author/funder, who has granted bioRxiv a license to display the preprint in perpetuity. It is made available under aCC-BY 4.0 International license.

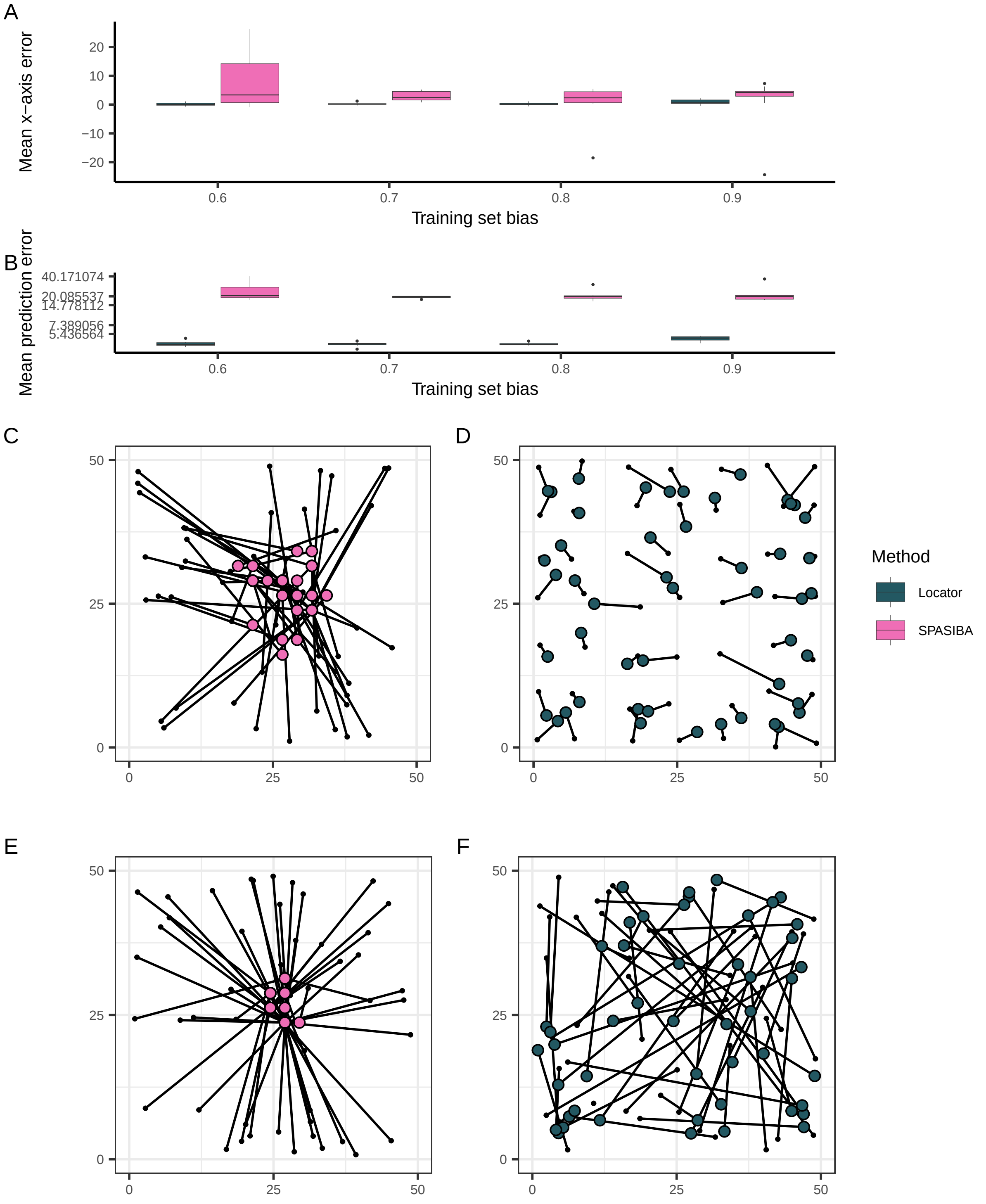

Figure S6: Predictions from biased training data using `SPASIBA` and `Locator`. Simulations had a per-generation dispersal rate of 1.0 units. (A) Mean x-axis error as a function of training set bias and (B) mean prediction error as a function of training set bias. (C, D) Example `Locator` and `SPASIBA` at $bias = 0.5$ and (E, F) $bias = 0.9$. True sample locations (black points) are connected to their predicted location (colored points).
